# Colchicine induction of ‘Old Blush’ 2n pollen for the hybridization and breeding of tetraploid rose

**DOI:** 10.7717/peerj.11043

**Published:** 2021-03-09

**Authors:** Shumin Gao, Yahong Sun, Yan Zhou

**Affiliations:** 1 College of Biological Sciences and Biotechnology, National Engineering Laboratory for Tree Breeding, Beijing Forestry University, Beijing, China; 2 Beijing Key Laboratory of Greening Plants Breeding, Beijing Institute of Landscape Architecture, Beijing, China

**Keywords:** ‘Old Blush’, 2n pollen, Colchicine, Induction, Function

## Abstract

Obtaining 2n pollen from the diploid Chinese old rose ‘Old Blush’ through artificial induction is one important means of hybridizing and breeding modern tetraploid roses. We used colchicine-induced 2n pollen to assess normal viability during hybridization and fructification. The results showed that the pollen mother cell had lagging chromosomes and parallel spindles at meiosis I stage, following which the 2n pollen was produced from dyads and triads with doubled chromosomes. We obtained 4.30% viable 2n pollen, which was significantly higher than the yield of the spontaneous 2n pollen (1.00%) using an optimal treatment combination of induction for 24 h with 0.50% colchicine. There was no significant difference between the external morphology of the induced 2n pollen and the spontaneous 2n pollen, whereas both types of 2n pollen possessed finer furrows, and fewer and smaller pores than the 1n pollen, and the external morphology of 2n pollen was more evolved. In terms of in vitro germination rate and pollen tube length, the induced 2n pollen did not differ significantly from the spontaneous 2n pollen. The survival rate of the floral buds was significantly decreased with increased colchicine concentration and treatment time.

## Introduction

‘Old Blush’, a rose in the Rosaceae family, is native to China and is the parent of some *Rosa hybrida* L. Rose is a popular flower with more than 30,000 varieties that are widely cultivated globally for their ornamental, edible, and medicinal applications (He, Yang & Liu, 2017). The ploidy of original rose plants, the ancestors of *Rosa hybrida*, is of various ploidy levels varies from diploid to octoploid. Modern rose has highly heterozygous genetic characteristics and has been created through tens of thousands of repeated hybridizations between Chinese and European rose species (Erlanson, 1938), involving bud mutations, mutagenesis, and molecular breeding for traits favorable for flower color, potpourri, thorns, disease resistance, and stress resistance. Hybridization is common in rose breeding and has a long application history and creating a very large seasonal population (Vries & Dubois, 2001). One main issue in cross-breeding is that the hybrid genetic background of traditional varieties is narrow, and the utilization of genetic resources tends to be saturated after long-term breeding. Genetic information among different species or distant relatives can result from distant hybridization, but is often associated with problems such as low affinity and poor fertility (Feng, Chang & Cong, 2009; Sun & Zhao, 2003). In modern rose breeding, a large gap still exists in China, and the number of new Chinese rose varieties contributed only 4% of the internationally registered varieties by 2016 (He, Yang & Liu, 2017).

‘Old Blush’ (2*n* = 2*x* = 14) is a diploid rose variety with excellent traits, including a long flowering season, which made it popular in the summer flowering season in Europe when it was introduced into the UK in 1793. Modern ornamental roses are mostly tetraploid with low hybrid affinity when crossed with the diploid rose, often resulting in infertile triploid offspring, which hinders breeding (Zlesak & Thill, 2005; Sugimoto et al., 2010). Using 2n pollen from the diploid rose for hybridization is one means of creating polyploids and can render diploid and tetraploid rose varieties able to hybridize, which constitutes a feasible strategy for solving infertility issues (Crespel et al., 2002; Crespel, Ricci & Gudin, 2006). 2n pollen production in roses has been reported by Crespel, Ricci & Gudin (2006) through obtained tetraploid offsprings. The percentages of 2n pollen and colchicine-induced 2n pollen had not reported. It has been reported that high temperatures or certain chemical reagents can cause microspore mother cell meiosis abnormalities, Including chromosome separation failure (chromosomal adhesion or backward chromosomes) and spindle abnormalities (parallel, fused, and tripolar spindles) (Liao et al., 2016; Li et al., 2017; Wang et al., 2017a; Wang et al., 2017b). Of these, parallel and fused spindles, and premature cytokinesis guide the formation of dyads, and the tripolar spindles create the tetrads during the tetrad period (Sun et al., 2011; Wu et al., 2011; Zhang & Kang, 2017), eventually leading to the formation of 2n pollen. At present, chemical mutagenesis can successfully induce somatic cell doubling to obtain homologous polyploids; for instance, the induction of diploid rose stem segments with oryzalin to obtain 44.4% autotetraploids (Allum, Bringloe & Roberts, 2007). The highest induction rate of three rose cultivars reached 66.7% using oryzalin, trifluralin, and amiprophos-methyl (APM) (Pegah et al., 2008). Since autopolyploid has not undergone the integration of repeated genomes, sterility often occurs in crosses. Autotetraploidy is a simple doubling of the diploids, whereas the 2n pollens (FDR type, first-division restitution), which double during meiosis I stage, have about 80% heterozygosity due to parental inheritance (Crespel et al., 2002; Xiao et al., 2007; Lü et al., 2017) and a higher utilization value. The chemical induction of 2n pollen has not been reported yet in *Rosa*, but several recent studies have researched the hybridization of 2n pollen in horticultural plants. Microtubule inhibitors, such as colchicine, have been used in the induction of 2n pollen in begonia, carnation (*Dianthus caryophyllus*), kiwifruit (*Actinidia chinensis*), tulip (*Tulipa* sp.), lily (*Lilium* sp.), and other species (Barba-Gonzalez et al., 2004; Okazaki et al., 2005; Dewitte et al., 2010; Wu et al., 2014; Zhou et al., 2015).

Using chemical reagents to induce 2n pollen is a common means of increasing 2n pollen production. The factors associated with the induction of 2n pollen relate to whether the pollen possesses normal viability, how to improve and enhance the induction rate, as well as the characteristic changes in the pollen based on the morphology. At present, many scholars have studied the germination rate and ultrastructure of 2n pollen induction in palynology. Zhao et al. (2015) studied the pollen morphology of six rose species, including the single-flowered yellow thorn, under a scanning electron microscope. The normal pollen of each variety possessed a three-holed ditch, and the polar view of the pollen was tri-circular at the equator. The equatorial surface was a long ellipse exhibiting a 1≃2 germinal furrow. These research results provide an important theoretical basis for the study of the germplasm resources of Rosaceae. The evolution and genetic characteristics of 2n pollen morphology were also researched in the aforementioned study. In this experiment, once the developmental process of ‘Old Blush’ pollen mother cells had been elucidated, we used colchicine to induce 2n pollen and assessed the viability during hybridization and fructification. The morphological characteristics of the induced 2n pollen and spontaneous 2n pollen were analyzed and evaluated for cross-breeding by scanning electron microscopy (SEM), which provides a basis for the use of ‘Old Blush’ as a genetic resource.

## Materials & Methods

### Plant material

Both the ‘Old Blush’ (2*n* = 2*x* = 14) and ‘Orange Fire’ (2*n* = 4*x* = 28) roses used in the experiment were collected from the Beijing Institute of Landscape Architecture (Beijing, China). The experiment was conducted from December 2015 to April 2018. Pollen mother cell (PMC) development observation and stamen materials used for colchicine induction were derived from the greenhouse cultivated perennial ‘Old Blush’, from which 2n and 1n pollens were collected from April to May every year. The female parent, a tetraploid modern rose ‘Orange Fire’, was grown in an open field.

### Colchicine induction of 2n pollen and its ultrastructural features

#### The relationship between the floral bud size and the development stage

We divided the pollen development process into five stages according to the floral bud morphology and the corresponding PMC development and meiosis characters. A total of 30 floral buds of different sizes from each development stage were collected from three plants. The length and width of the floral buds were measured. Samples were fixed in a modified formalin-acetic alcohol (FAA) solution for more than 12 h and stored at 4 °C. The pollen development stages were ascertained using the general squash technique, and the three anthers in each floral bud were observed by optical microscope. We observed at least 200 pollens in each anther and determined the relationship between both the floral bud size and the development stage. We focused on the chromosomal abnormalities of the PMCs during the meiotic period, such as chromosome bridges, parallel spindles, and doubling of the chromosomes.

#### Colchicine induction of 2n pollen

Colchicine induction of 2n pollen in ‘Old Blush’ was carried out from mid-April to the end of May. A metaphase developmental stage of bud was selected for colchicine induction. The colchicine with concentration of 0% (controls or background values), 0.25%, 0.50%, 0.75%, and 1.00% (W/V) were prepared. The treatment time was 0 h, 24 h, 48 h and 72 h, and each treatment included 20 floral buds. We injected 0.2 mL colchicine solution into the center of the floral bud using a disposable syringe and then immediately wrapped the floral bud in cotton wool soaked with the same concentration colchicine solution, which was then fastened with sealing film for 24 h or longer. The same colchicine solution was added to the cotton wool every 24 h. The controls were injected similarly, lacking colchicine in the application solution, but with similar pH and osmotic potential. After treatment, the cotton wool was removed. The survival rate of the floral buds in the different treatments was calculated a week later. About two weeks later, the spontaneous, colchicine-treated 1n, 2n pollen were collected from the floral buds. The percentage of spontaneous, treated 1n, 2n pollen in the different treatments was investigated by staining with aceto-carmine, and 1n, 2n pollen vitality was assessed by staining with Alexander dye solution followed by observation under optical microscope. The percentage of spontaneous 1n or 2n pollen is out of the total 1n + 2n pollen in buds from control conditions. The percentage of treated 1n or induced 2n pollen is out of the total 1n + 2n pollens in the bud of a colchicine-induced treatment, and then subtract the controls or background values, i.e., the percentage of 1n or 2n pollen is out of the total 1n + 2n pollens in the bud with same time plus a 0% colchicine concentration treatment. The percentage of viable 1n or 2n pollen is out of the total 1n or 2n pollen in the bud from control conditions or a colchicine-induced treatment. The percentage of induced viable 2n pollen is out of the total treated or induced 1n + 2n pollens in the bud of a particular treatment, which come from the percentage of induced 2n pollen times the percentage of viable 2n pollen. The experiment was repeated three times. The pollen diameter was measured using Digimizer software (a product of Medcalc Software, a developer of medical and statistical software, info@medcalc.org), and referenced the 2n pollen diameter was 1.3 times that of the 1n pollen from our published data (Gao et al., 2019). The optimal concentration and treatment time were comprehensively evaluated.

#### Ultrastructural observation of the pollen

To study the ultrastructure, 30 spontaneous 2n pollen and 30 colchicine-induced 2n pollen were fixed in 3% fresh glutaraldehyde for 24 h at 4 °C and then washed three times with phosphate buffer (pH = 6.8) and dehydrated in t-butanol solution at a series of concentrations. The samples were freeze-dried and then sprayed gold with an ion sputter coating apparatus. The outer wall ornamentation of spontaneous 1n and 2n pollen, treated 1n pollen and induced 2n pollen grains, such as ridge, furrow and aperture, were observed and distinquished, and their polar axis and equatorial axis lengths were measured under a scanning electron microscope (TM3000, Hitachi, Japan).

### Characteristics of the colchicine-induced 2n pollen

Compared with spontaneous 1n and 2n pollen, the in vitro germination and growth characteristics of the colchicine-induced 2n pollen was observed by microscope. After 12 h of dark culture in vitro germination medium with 10% sucrose, boric acid and calcium chloride at 20 °C, germination rate was counted and the average length of the pollen tube was measured. Three traits such as extreme view, equatorial view and surface decoration of the spontaneous 1n, 2n pollen, and induced 2n pollen were measured by three views, and the amount of pollen in each view was higher than 100 pollens. The pollen tube length was measured and averaged 30 pollens.

The 2n pollen and induced 2n pollen germination on the stigma and growth in the style of ‘Orange Fire’ were observed under a fluorescence microscope Leica DM 6000B. We observed and measured the pollen germination rate and the average length of the pollen tube in vitro using 10% sucrose plus boric acid and calcium chloride medium. The samples were placed in the dark at 20 °C for 6 h and observed later under a microscope.

Spontaneous 2n pollen and induced 2n pollen of ‘Old Blush’ were pollinated on the stigma of about 50 ‘Orange Fire’ floral buds that had been castrated and bagged, and 20 pollinated floral buds were developed and used for calculating the fructification rate. We collected 5 pollinated floral buds from each pollinated stage of 0 h, 4 h, 24 h and 72 h, fixed the pistil with Carnot’s fixative for 24 h, transferred them to 70% alcohol, and then stored them at 4 °C. They were then made transparent and softened with 0.8 mol/L NaOH solution and stained with 0.1% aniline blue solution according to the method of Zhou et al. (2012). The spontaneous 2n pollen and induced 2n pollen germination on the stigma and growth in the style of ‘Orange Fire’ were observed under a fluorescence microscope (Leica DM 6000B). The experiment was repeated three times.

### Identification of ploidy by flow cytometry for the chromosomes of the stemtips

About 1.0 g of fresh leaves of each plant was used. The samples were washed with distilled water, dried with filter paper, and placed on a chilled Petri dish onto whichpre-cooled LB01 lysis buffer approximately (1–2 mL) was added, and the leaves were rapidly chopped once with a sharp blade. The samples were immersed in thelysis buffer during the entire process in order to efficiently dissociate the nuclei. The lysis buffer was absorbed by the samples from the dish and filtered intoa 1.5 mL eppendorf tube through a 400 µm mesh filter membrane and then placed in a refrigerator and incubated for 5 min at 4 °C. The samples were then centrifuged at 1,500 r/min for 5 min at 4 °C. Then, 100 µL of chilled lysis buffer and 150 µL of chilled PI (Propidium Iodide) dye were added to each sample after discarding the supernatant. The samples were then stored in the dark in a refrigerator and stained for 10 min at 4 °C. The samples were then transferred to the sample tubes, using flow cytometry (FCM) (BD Company Accuri C6 flow cytometry), we detected and collected 5000–10,000 particles. The known ploidy of ‘Old Blush’ was used as the external standard for ploidy, and the ploidy samples were determined by comparing the intensity of their fluorescent signals.

### Molecular identification of genuine hybrids of F1 offspring using SSR molecular markers

Genomic DNA was extracted using a DP320 plant genome extraction kit (Tiangen Bioch. Tech. Com., Beijing, China). The samples were separated by electrophoresis on 0.8% (w/v) agarose gels. The quality and concentration of DNA was assessed using a UV spectrophotometer. These were then diluted to 25 ng/µL and stored in a refrigerator at −20 °C during our experiments.

Using the genomic DNA as a template in the SSR experimentand the female parents of ‘Orange Fire’ and the male parents of ‘Old Blush’, A total of 21 SSR markers were surveyed for parental polymorphism between female parents of ‘Orange Fire’ and male parents of ‘Old Blush’ (Table S1) (Yu, 2015). The polymorphic SSR markers were screened on the F1 hybrid progeny. The primer combinations were designed, screened, and synthesized by the Beijing RuiboXingke (http://www.ruibiotech.com) Biological Technology Ltd., Co..

Each 25 µL PCR mixture consisted of 12.5 µL 2 ×Taq PCR Master Mix, 1 µL of a primer solution, 1 µL template solution, and 10.5 µL of ddH_2_O. The following PCR conditions were used: initial denaturation at 94 °C for 5 min, followed by five cycles of denaturation at 94 °C for 1 min, annealing at 35 °C for 1 min, extension at 72 °C for 1 min, and then 30 cycles of denaturation at 94 °C for 1 min, annealing at 50 °C for 1 min, extension at 72 °C for 1 min, and a final extension at 72 °C for 10 min. The samples were separated by electrophoresis on 2% (w/v) agarose gels. Each gel electrophoresis was runat a constant voltage of 150 V at 20 °C for 30 min and then observed and photographed using a gel imaging system.

### Statistical analysis

In this study, the experiment was carried out following a completely randomized design (three replications). Data were expressed as the means ± SD, and analysed using ANOVA by the SPSS 17.0 (SPSS Inc., Chicago, IL, USA). All data were performed using Origin 8.0 software (OriginLab Corp., https://www.originlab.com/).

## Results

### Colchicine-induced 2n pollen of ‘Old Blush’

#### Correlations between floral bud size and pollen development stage in ‘Old Blush’

**Figure 1:**
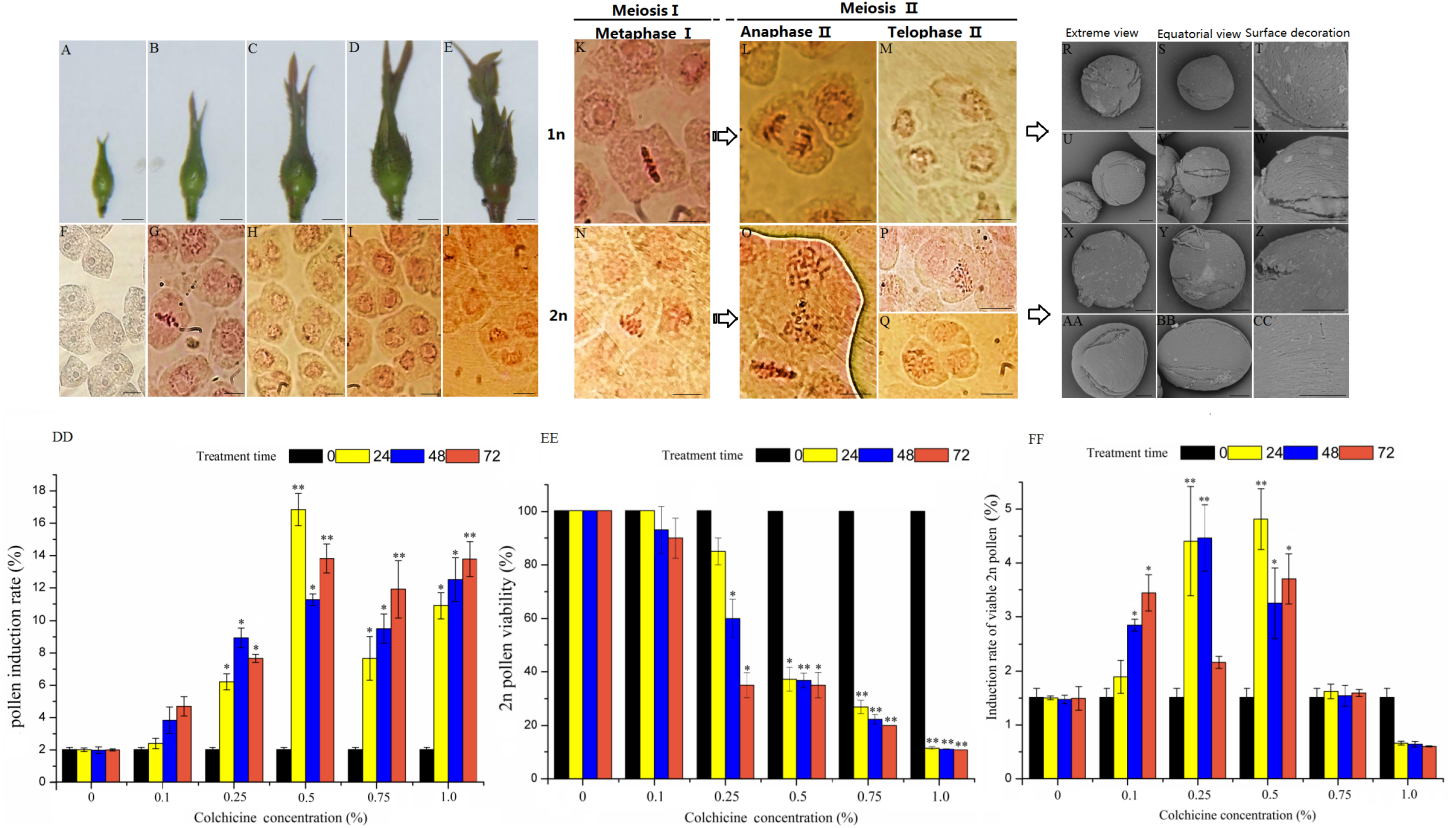
Pollen characteristics at different development stages and 2n pollen induction in the floral buds of ‘Old Blush’. (A–E) Five floral bud stages of ‘Old Blush’, bar = 5 mm. (F) PMC at floral bud stage 1; almost all are polygonal and the nucleoli are distinct; (G). PMC at floral bud stage 2, the early stage in meiosis; (H) PMC at floral bud stage 3; most are dyad; (I) PMC at floral bud stage 4; the dyads and tetrads are roughly half of the total pollens, including a small amount of triads; (J) PMC at floral bud stage 5; meiosis is complete and all cells are tetrads, (F–J). bar = 10 µm. (K) Interim I; (L) Late II; (M) Terminal II (quadruplex); (N) Lagging chromosomes of metaphase I; (O) Parallel spindle (pole view); (P) Diploid; (Q) Triad, (K–Q) bar = 5µm. (R–T) 1n natural pollen; (U–W) Colchicine-treated 1n pollen; (X–Z) Natural 2n pollen; (AA-CC) Colchicine-inducted 2n pollen; (R, U, X, AA). Pollen pole view (2000 ×5KV); (S, V, Y, BB) Pollen equatorial view (2000 × 5KV), bar = 10 µm; (T, W, Z, CC) Pollen surface ornamentation (8000 × 5KV),bar = 5 µm. (DD–FF) Colchicine-induced 2n pollen of ‘Old Blush’ under different treatments, (DD) Induction rate of 2n pollen; (EE) Induced 2n pollen vitality; (FF) Induction rate of viable 2n pollen.“*”significance testing, *P* < 0.05; “**”significance testing, *P* < 0.01, analysis of significant differences in different treatment concentrations.

**** Pollen mother cells begin to develop at the same time as the floral buds begin growth in ‘Old Blush’. We divided the pollen development process into five stages according to the floral bud morphology and the corresponding PMC development and meiosis characters (Figs. 1A–1J, Table 1). At floral bud stage 1, the tender bud was tightly wrapped by the tender leaves and sepals (Fig. 1A). The PMC had not begun to divide, showing polygonal and distinct nucleoli (Fig. 1F). At floral bud stage 2, the floral buds gradually enlarged and remained tender green, and the bracts became elongated (Fig. 1B). The PMC was in the prophase of meiosis when the nucleolus disappeared and the nucleus became enlarged, and the division period of the cell could be identified by chromosome arrangement or spindle morphology (Fig. 1G). At floral bud stage 3,the color of the flower buds had intensified (Fig. 1C), most of the cells were dyads, and the remainder were tetrads or single cells at incomplete meiosis I (Fig. 1H). At floral bud stage 4, the color of the torus and the bracts had intensified, and the torus and flower buds were both enlarged (Fig. 1D). Both the dyads and tetrads were about half as numerous as the pollen, and cells dividing at different stages of meiosis II could be observed. A small number of single cells had not yet completed meiosis I (Fig. 1I). At floral bud stage 5, the ovaries and perianth were satiated, the floral buds started becoming red (Fig. 1E), most of the cells were tetrads at the end of meiosis II (Fig. 1J), and the PMC had completed meiosis. The pachytene stage was the most efficient stage of 2n pollen induction, but the meiosis process in ‘Old Blush’ pollen is highly asynchronous. Different pollens in an anther are often in different stages, and thus we can induce 2n pollen during floral bud stage 2 when most pollens are still developing at meiosis I stage.

**Table 1:** The correlation between floral bud size and pollen development stage in ‘Old Blush’.

No.	Floral bud length (mm)	Floral bud width (mm)	Floral bud morphology	Floral bud development stage	Cell morphology
1	5.84 ± 0.41	3.24 ± 0.11	Floral bud emergence	Prophase of meiosis	Nucleoli evident
2	7.76 ± 0.74	4.24 ± 0.04	Length oval-shaped	Early phase of meiosis	Nucleolus disappears and chromosomes or spindles are visible
3	10.12 ± 0.64	5.28 ± 0.09	Floral bud expension, and sepal, flower stalks arise glandular hairs	Meiosis I phase	All cells are dyad
4	13.26 ± 0.56	6.28 ± 0.08	Fully sturdy flower buds	Meiosis II phase	One half of each dyad and tetrad
5	13.45 ± 0.68	7.13 ± 0.17	Floral bud is reddening	Metaphase	About 90% of the tetrads

The chromosomes were clearly observed during each vigorous period of microspore meiosis in ‘Old Blush’. The nucleus disappeared and seven pairs of chromosomes were arranged in a two-pair confluence state in the cell at prophase I stage. The chromosomes were concentrated in the cell center equatorial plate at metaphase I stage (Fig. 1K). The homologous chromosomes were separated and the spindles moved towards the cell poles at the anaphase I stage. The spindle arrived at the poles and the chromosomes were restored to the nucleus at telophase I stage. Chromosomes rearrangement showed seven chromosomes containing sister chromatids at prophase II. The chromosome also moved toward the poles under the spindle traction at metaphase II (Fig. 1L) and anaphase II (Fig. 1M), eventually forming a tetrad. There was an abnormal spindle phenomenon in ‘Old Blush’, which eventually led to 2n pollen formation. The abnormal chromosomes were not concentrating on the equatorial plate and lagging during meiosis metaphase I (Fig. 1N), eventually leading to unequal chromosome division and the spindle not moving in opposite directions (Fig. 1O). In addition, we observed a dyad with a doubled chromosome set (Fig. 1P) and a triad composed of 2n cells and two normal 1n cells in the tetrad period (Fig. 1Q). These results indicated that the abnormal chromosomal behavior led to 2n pollen production. However, the proportion of 2n pollen was very low under natural conditions during the meiosis stage, and thus it is difficult for it to be used in hybridization, thus requiring artificially-induced 2n pollen.

**Table 2:** Morphological characteristics of natural 1n and 2n pollen and induced 2n pollen of ‘Old Blush’ observed by SEM.

Pollen type	Polar axis (µm)		Equatorial axis (µm)		P/E	Pollen shape (equatorial view)	Pole view	Germinal aperture	Furrows of pollen outer ornamentation
Natural 1n pollen	21.00–26.00	23.62	24.00–24.30	24.17	0.98	Near sphere	Tilobated rotund	3	With narrow and intersecting ridges, and deep and crossover furrows containing a high–density large aperture
Natural 2n pollen	27.60–34.20	32.20	29.90–32.70	30.69	1.05	Near sphere	Tilobation rotundity	3	With wide and inconspicuous ridges and fine and inconspicuous shallow furrows containing a low-density small aperture, there are no apertures near the pole
Induction 1n pollen	20.60–25.70	23.50	24.10–25.60	24.70	0.95	Near sphere	Tilobation rotundity	3	With narrow and distinct ridges and dense and deep furrows containing a low-density small aperture
Induction 2n pollen	29.70–40.30	33.30	30.20–36.20	32.45	1.03	Near sphere	Tilobation rotundity	3	With wide and inconspicuous ridges and sparse and shallow furrows containing a high-density aperture near the equator and low-density aperture near the pole

##### Scanning electron microscopy

The morphological characteristics between the treated 1n and induced 2n pollens and natural 1n and 2n pollens of ‘Old Blush’ were observed and compared (Table 2, Figs. 1R–1CC). The equatorial view of the four pollens indicated a near-spherical shape after imbibition, and the polar view indicated a three-lobed circular shape with three-holed grooves with furrows and apertures of different widths and depths among them. The pollens were N3P4C5 type. Both the natural and induced 2n pollens were larger in size than the 1n pollen. The polar axis of the natural 1n pollen was 23.62 µm (21.00–26.00 µm) and the equatorial axis was 24.17 µm (24.00–24.30 µm). The polar axis of the natural 2n pollen was 32.20 µm (27.60–34.20 µm) and the equatorial axis was 30.69µm (29.90–32.70 µm). The polar axis (2x) of the treated 1n pollen was 23.50µm (20.60–25.70 µm) and the equatorial axis was 24.70µm (24.10–25.60 µm). The polar axis (2x) of the induced 2n pollen was 33.30 µm (29.70–40.30 µm) and the equatorial axis was 32.45 µm (30.20–36.20 µm). The outer wall ornamentation of the four types of pollen differed. Natural 1n pollen has wide ridges, and deep furrows containing a high-density large aperture (Figs. 1R–1T); The treated 1n pollen has wide ridges and deep furrows containing alow-density small aperture (Figs. 1U–1W). Natural 2n pollen has narrow ridges and shallow furrows containing a low-density small aperture, there are no apertures near the pole (Figs. 1X–1CC).The induced 2n pollens has narrow ridges and shallow furrows containing a high-density aperture near the equator and low-density aperture near the pole (Figs. 1AA–1CC). The induced 2n pollen has wider ridges and shallower furrows than the natural 2n pollens, but the difference is not significant. Compared with 1n pollens, 2n pollens showed narrower ridges, shallower furrows and smaller aperture density, with evolutionary characteristics.

The colchicine concentration and treatment times affected the induction rate of ‘Old Blush’ 2n pollen (Figs. 1C–1D, Table S2). The optimum composition of colchicine induction was 0.50% for 24 h, during which the highest induction rate of 15.80% was obtained. The induction rates of the different concentrations of colchicine were significantly different based on the variance analysis, and there was no significant difference in the induction rates with different treatment times (Fig. 1E, Tables S2, S3). We proposed that there was no significant difference in 2n pollen induction rates based on a comprehensive assessment of viability.

**Figure 2:**
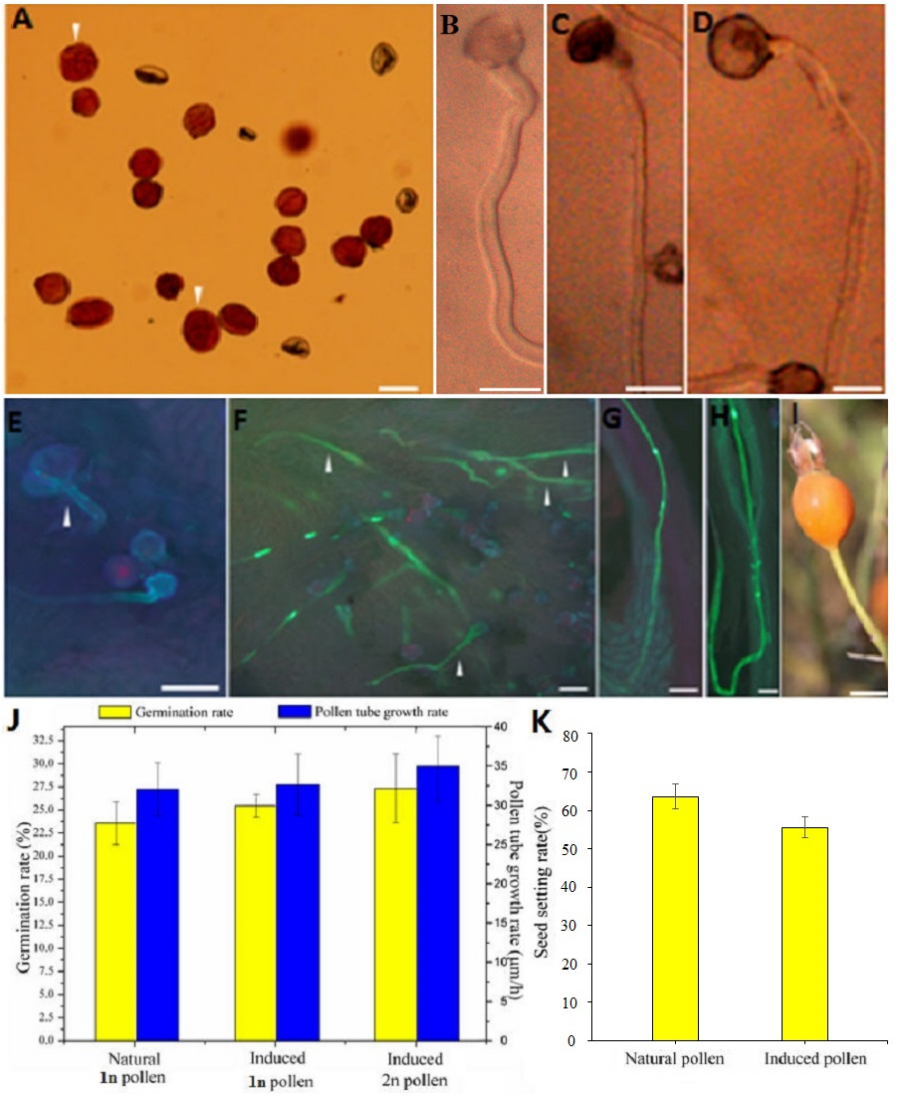
Germination, growth, and fructification characteristics of the colchicine-induced 2n pollen. (A) The colchicine-induced‘Old Blush’ 2n pollen viability was measured by Alexander staining. The arrows indicate viable 2n pollen; (B) natural 1n pollen germination in vitro; (C) colchicine- treated ‘Old Blush’ 1n pollen germination in vitro; the natural and treated 1n pollen grains could not be distinquished on the base of B and C. (D) colchicine-induced ‘Old Blush’ 2n pollen, (A–D) bar = 25 µm; (E) colchicine-induced ‘Old Blush’ 2n pollen germinating on the stigma 4 h after pollination; F. colchicine-induced ‘Old Blush’ 2n pollen germinating on the stigma 24 h after pollination; (G) Natural 1n pollen tuber grew into the ovary 72 h after pollination; (H) colchicine-induced ‘Old Blush’ 2n pollen germinating on the stigma 72 h after pollination. The arrows indicate a viable 2n pollen tuber, E-H.bar = 50 µm. (I) fructification of ‘Orange Fire’ hybridized (♂) with colchicine-induced ‘Old Blush’ 2n pollen (♂), bar = 1 cm; (J) Germination rate and pollen tube growth rate of ‘Old Blush’ natural 1n pollen, treated 1n pollen, and induced 2n pollen for 12 h in vitro. (K) Seed setting rate of ‘Orange Fire’ hybridized (♂) with colchicine-induced ‘Old Blush’ 2n pollen (♂).

While the 2n pollen was induced with 1.00% colchicine at different times, the survival rate was very low of 0.80%-1.60% (Fig. 1D, Table S2) although the induction rate was as high as 9.90%-12.80% (Fig. 1C, Table S2). However, the 2n pollen viability was much higher 25.00%-83.00% (Fig. 1D, Table S2) at a low colchicine concentration of 0.10%-0.25%, although the induction rate was not higher 1.40%–7.90% (Fig. 1C, Table S2). Our results proposed that a colchicine concentration of 0.50% plus 24 h of treatment resulted in a high induction rate of 4.30% in the 2n pollen (Table S2).

### Biological functioning of the colchicine-induced ‘Old Blush’ 2n pollen

#### In vitro germination characteristics of the colchicine-induced 2n pollen

Compared with the natural 1n and 2n pollen, the colchicine-treated 1n and colchicine-induced 2n pollen germinated normally, and its pollen tube was sturdier (Figs. 2B–2D). The 1n pollen germination rate was decreased from 84.20% to 78.10%, and the 2n germination rate was decreased from 100.00% to 27.20% (Table S2). There was no significant difference in the growth rate of pollen tube between the natural pollen and treated 1n or induced 2n pollen, whereas there was significant difference between the natural pollen and induced 2n pollen and a slight difference between the natural pollen and treated 1n pollen in the germination rate (Fig. 2J).

##### Germination, growth, and cross-breeding characteristics of the colchicine-induced ‘Old Blush’ 2n pollen after pollination

**Figure 3:**
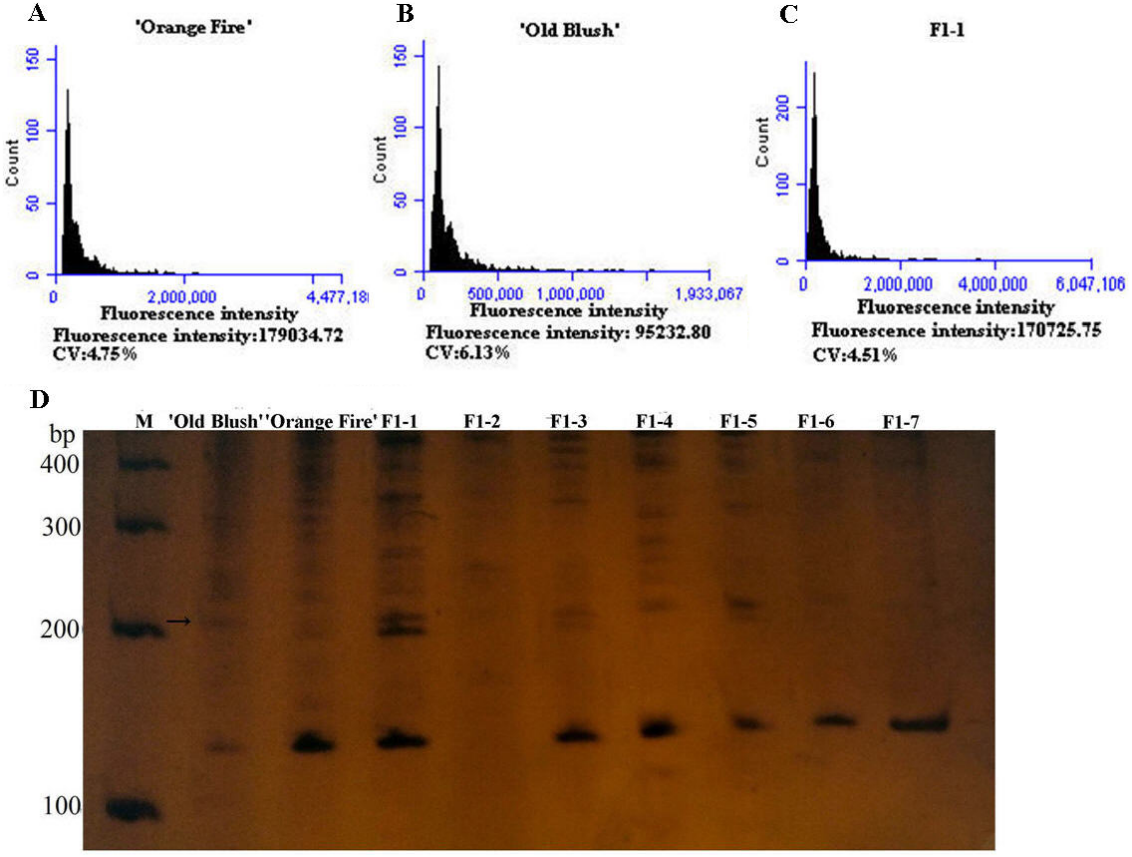
Genetics of ploidy characteristics of ‘Orange Fire’ × ‘Old Blush’ and results of SSR for the F1 of ‘Orange Fire’ × ‘Old Blush’. (A) Chromosome ploidypicture of ‘Orange Fire’ detected by flow cytometry with a fluorescence intensity of 179,034 (2*n* = 4*x* = 28). (B) Chromosomeploidy picture of ‘Old Blush’ detected by flow cytometry with a fluorescence intensity of 95,232 (2*n* = 2*x* = 14). (C) Chromosome ploidy picture of No.1 F1 from ‘Orange Fire’ × ‘Old Blush’ detected by flow cytometry with a fluorescence intensity of 170,725 (2*n* = 4*x* = 28). (D) Results of SSR for the F1 (No.1–7) of ‘Orange Fire’ × ‘Old Blush’. The black arrows indicate the location where the specific band of a male parent and F1 No.1, 3, 5 appeared.

The diameter of the colchicine-induced 2n pollen was 1.3 times larger than that of the 1n pollen. The red and near-round induced 2n pollen was viable, whereas the colorless and empty pollen had no viability based on the Alexander staining of the 2n pollen (Fig. 2A). Four hours after pollination, the 0.50% colchicine-induced 2n pollen (for 2 d) germinated normally on the stigmata of ‘Orange Fire’, and the 2n pollen tube was wider than that of the 1n pollen using aniline blue staining (Fig. 2E). In our previous results, there was a significant positive correlation between pollen tube width and pollen diameter. Twenty-four hours after pollination, a large number of induced 2n pollen tubes had grown into the stigma (Fig. 2F). Seventy-two hours after pollination, most of the induced 2n pollen tubes were similar in growth to the natural 1n pollen (Fig. 2G) and the 2n pollen had grown into the ovary (Fig. 2H). The seed setting rate of natural pollen pollination was 63.60%, and the seed setting rate of induced 2n pollen pollination was 55.60% in autumn (Table S8). There was no significant difference in the seed setting rate between induced 2n pollen and natural pollen pollination (Figs. 2I, 2K). The ploidy identification of the parents and F1 offspring in hybrid combinations showed a 100% precision rate by flow cytometry (Figs. 3A, 3B, 3C). F1 tetraploid generation by SSR identification indicated that 2n gametes of ‘Old Blush’ involved in F1 tetraploid generation (Fig. 3D). The RW55C6 primer was amplified in the parents and generated specific parental bands of high reproducibility with one target band size of about 205bp. The RW55C6 primer in the offsprings also amplified good polymorphic bands. If the offspring has specific bands of both parents, then we consider that the offspring is heterozygous. It is normal for 205bp bands of paternal ‘Old Blush’ independently. The F1 No.1, 3, 5 hybrids detected using this primer in ‘Orange Fire’ × ‘Old Blush’ constitute true hybrids. Therefore, the colchicine-induced 2n pollen has a similar biology and function to the natural 1n and 2n pollen and can thus be used for cross-breeding.

**Figure 4:**
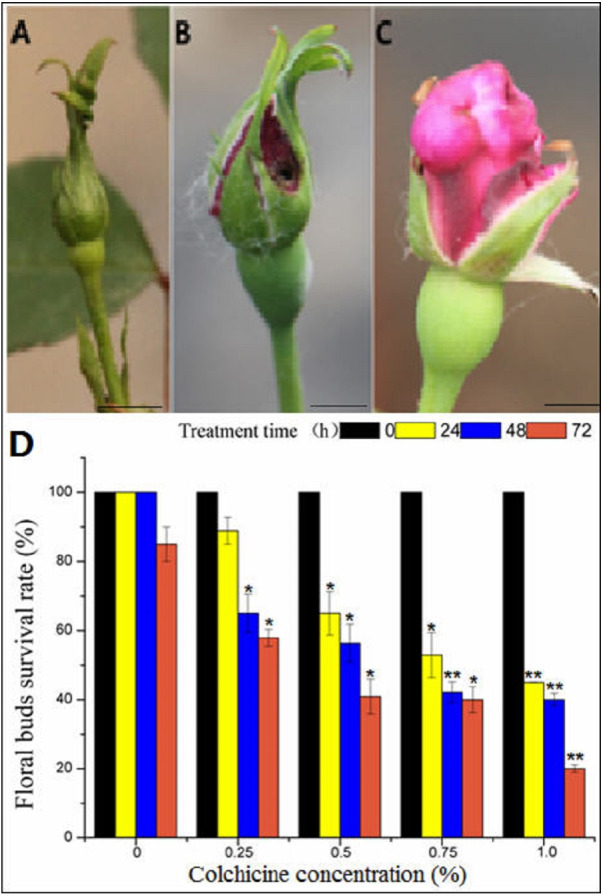
The toxic effect of colchicine on ‘Old Blush’ floral buds. (A) Floral bud before treatment; (B) a control floral bud treated with water for two weeks; (C) a floral bud treated with colchicine for two weeks, (A–C). bar = five mm. (D) Survival rate of ‘Old Blush’ floral buds.“*”significance testing, *P* < 0.05; “**”significance testing, *P* < 0.01, analysis of significant differences in different treatment concentrations.

#### Survival rate of the ‘Old Blush’ floral buds treated with colchicine

In general, the flower buds were collected at mature stage of pollen after removal of cotton wool two weeks later (Fig. 4A, Table S2), most of the floral buds of the control group injected with distilled water developed normally, indicating that there was no damage to the floral buds (Fig. 4B). In contrast, the floral buds injected with colchicine demonstrated severe symptoms of poisoning, such as sepal atrophy, dried-up tips, and deformed petals, and the amount of pollen was greatly decreased (Fig. 4C). The floral buds also fell off during the floral bud development process, indicating severe colchicine drug damage. Different treatments produced different survival rates (Fig. 4D). The survival rate of the floral buds injected with distilled water was 100% for 24 h and 48 h, and the survival rate decreased to 85% at 72 h (Table S2), indicating that a long period of being wrapped in wet cotton will lead to floral bud death. With the increase in concentration and treatment time, the survival rate of the floral buds decreased. The survival rate of the floral buds differed significantly at different concentrations and different treatment times based on the two-factor analysis of variance (ANOVA) (Fig. 4D, Table S2, S4, S5, S7).

## Discussion

**Figure 5:**
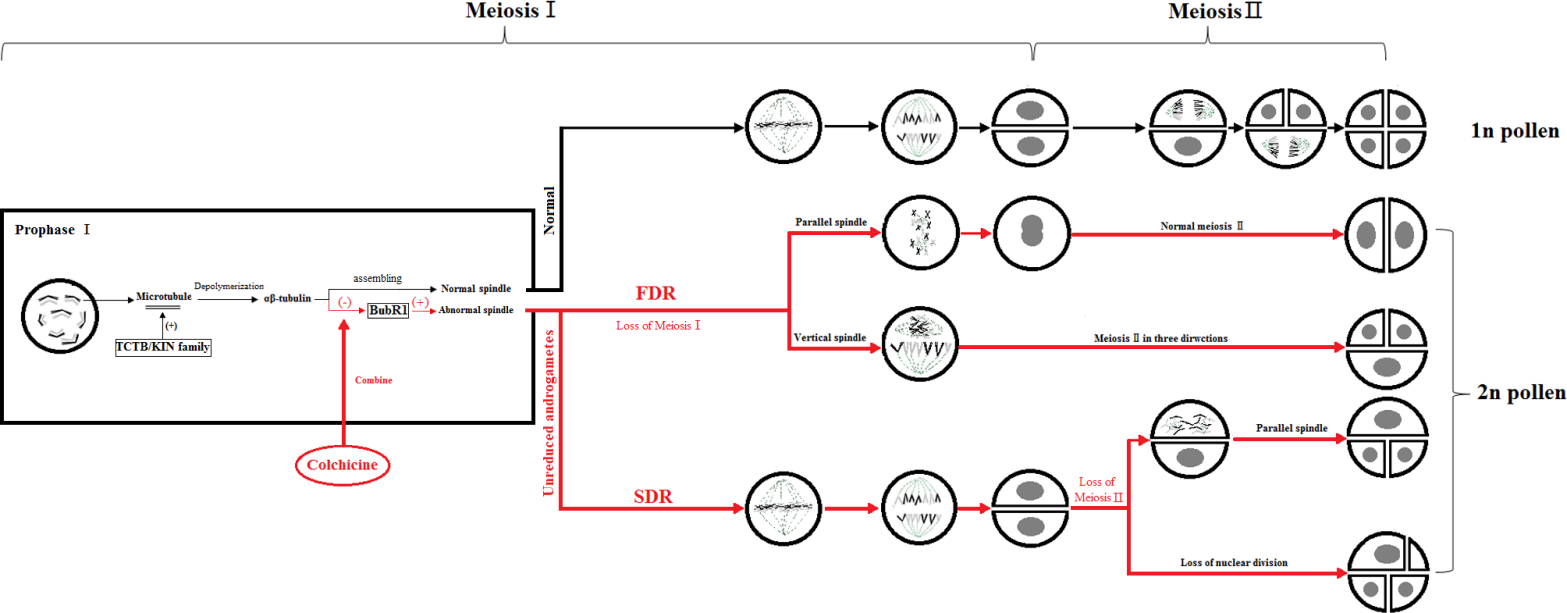
Colchicine induced 2n pollen pattern. The red part means the process of 2n pollen production induced by colchicine, “(-)” means the inhibitory effect, “(+)” means the promoting effect, “ □”means protein.

The key to the chemical induction of ‘Old Blush’ 2n pollen involves finding a balance between the doubling and fatality of the pollen by controlling the chemical agent concentration and treatment time to obtain the highest induction efficiency. During mitosis, colchicine binds to α-tubulin and β-tubulin produced by microtubule depolymerization in the cell, preventing the polymerization of microtubules, thus inhibiting the formation of spindle, preventing chromosome segregation, promoting DNA replication and preventing cell division. Some genes related to colchicine regulating male gamete meiosis were screened from the ‘Old Blush’ pollen transcriptional database of our research group. TCTB (translationally-controlled tumor protein homolog) was regulating microtubule depolymerization during cell cycle mitosis. The microtubule was depolymerized into α -tubulin and β -tubulin (Fig. 5). BUBR1 (mitotic spindle checkpoint protein) is a checkpoint gene for mitotic spindle assembly, participates in the initiation of cytokinesis and DNA replication, and regulate the process of DNA replication. Evidence suggests that spindle disruptors, such as colchicine, cause spindle detection points to be abnormal, with immature, abnormal, or stranded chromosomal cells that can continue to divide, leading to chromosome segregation abnormalities (Yan, 2004) (Fig. 5). Unlike the traditional kinesins, the members of the Kin I family are microtubule depolymerases (Fig. 5). It is assumed that these biomolecules can produce polar forces on the kinetochore, and play an important role during the medium term (Garcia, Koonrugsa & Toda, 2002). In the process of induced 2n pollen by colchicine, some abnormal spindles, such as parallel spindle, vertical spindle, fusion spindle and multipole spindle, will appear, which is related to the polarity and polar force of spindle distribution. It is speculated that colchicine may also act on the KIN protein family, affecting the generation of polar force, and result in the abnormal distribution of the spindle and eventually produce unreduced male gametes.

We observed that the floral buds in the meiosis I stage treated with 0.50% colchicine for 24 h exhibited a reduced induction rate and pollen viability. This was also observed at higher concentrations and longer treatment durations. Natural 2n pollen is produced from abnormal meiosis in the pollen mother cells, presenting dyads and triads in the tetrad period and abnormal spindle behavior. The pattern of 2n pollen formation in Chinese rose is genetically equivalent to the FDR mechanism (Crespel, Ricci & Gudin, 2006; Pécrix et al., 2011). When the floral buds of *Eucalyptus* (3.5–4.0 mm) were treated with 0.50% colchicine solution for 6 h, the highest yield of 2n pollen (28.7%) was obtained, indicating that floral bud development stage, colchicine concentration, and treatment time could affect the colchicine-induced 2n pollen (Yang et al., 2016). The induction of somatic cell doubling using several similar microtubule inhibitors in rose, such as colchicine treatment for 24 h, is sufficient to inhibit mitosis and produce tetraploid cells. Increasing the colchicine concentration or prolonging the treatment time may aggravate or superimpose the effect, resulting in multiploidy and mixed-ploidy cells. Thus, we were unable to obtain a higher doubling rate while the survival rate was decreasing (Allum, Bringloe & Roberts, 2007; Pegah et al., 2008). In our experiments, we also observed large pollens after 48 h of treatment, which may suffer similar effects. The associated processes might, thus, be quite complex and are worth investigating in the future.

Compared with natural 1n pollen, both the induced 2n pollen and natural 2n pollen are larger with more narrow furrow ornamentation and smaller, denser apertures, but with no apertures near the extremes. The induced 2n pollen had fewer apertures than the natural 2n pollen, but the furrow ornamentation was uniform. These results indicated that colchicine induction mainly affected the distribution and characteristics of the apertures in the pollen wall of rose, but had little effect on the furrow ornamentation. The results suggest that we could use SEM to identify 2n pollen from 1n pollen in mixed pollens. 2n pollen was originally identified based on the ploidy of the filial generation by flow cytometry and pollen size analysis. 2n pollen production is related to the genotype, and its function can be transferred to the next generation through the 2n gametes of the parents (Crespel, Ricci & Gudin, 2006). We observed that induced 2n pollen was near spherical, and the polar view indicated that it was three-lobed and round with three germination pores. Zhou, Wei & Wu (1999) suggested that cavernous ornamentation might be primitive in Rosaceae, with furrow ornamentation being a derived feature. Tian et al. (2018) found that high temperature sometimes affected the deposition of the outer wall ornamentation. Compared with natural 2n pollen, the surface ornamentation of the high-temperature-induced 2n pollen appeared to be long and with narrow furrows, but no significant difference between the natural 1n pollen and treated 1n pollen was observed.

Most polyploids in the natural world are produced from unreduced gametes, and the allopolyploids formed from unreduced gametes can combine diploid genetic resources via reproductive isolation (Wang et al., 2017a). Plant polyploidy enriches plant genetic resources and provides materials for variation. Polyploidy is often characterized by abundant phenotypic variation, larger organs, increased metabolite production, and enhanced stress resistance (Rim & Beuselinck, 1996). Producing polyploid plants through 2n pollen constitutes a breeding high-quality varieties. The induced activity of 2n pollen is the primary issue to consider for breeding. Artificial induction may increase the germination pores of 2n pollen into tetrahedrons, affecting the growth rate or even stopping the growth of 2n pollen, thus resulting in poor competition of the 2n pollen during breeding (Song et al., 2017). In vitro or in vivo, colchicine-induced ‘Old Blush’ 2n pollen demonstrated similar germination activity and growth rates to natural 2n pollen, and our findings corroborated previous studies (Tian et al., 2018). Increasing the competitiveness of 2n gametes will promote the creation of polyploid rose. The growth difference between 2n pollen and 1n pollen during germination requires further exploration.

In the plant polyploidization process, genes not only do double but also are subject to silencing, deletion, recombination, and transformation, leading the plant genome to acquire stable diploidization, and genes with different functions have different evolutionary rates. For example, the genes involved in transcription and signal transduction are preferentially retained, while the genes involved in the repairing and encoding of organelle proteins are easily lost (Adams & Wendel, 2005). Therefore, the application of 2n pollen in long-term breeding work cannot simply consider paternal genome diploidization. The genetic laws of the different traits need to be analyzed in order to achieve different breeding aims.

## Conclusions

Obtaining higher percentage normal viable 2n pollen from the diploid Chinese old rose ‘Old Blush’ through colchicines induction is one important means of hybridizing and breeding modern tetraploid roses. Only a limited number of colchicine-induced 2n pollen involved in polyploidy breeding have been characterized in detail.We obtained 4.30% 2n pollen, which was significantly higher than the yield of the spontaneous 2n pollen (1.00%) using an optimal treatment combination of induction for 24 h with 0.50% colchicine, and observed the external morphology of 2n pollen more evolved. The results of this study can contribute to the use of diploid genetic resources in rose breeding.

##  Supplemental Information

10.7717/peerj.11043/supp-1 Supplemental Information 1 Figures S1-S5

10.7717/peerj.11043/supp-2 Supplemental Information 2 Tables S1-S8

10.7717/peerj.11043/supp-3 Supplemental Information 3 Pollen characteristics at different development stages and 2n pollen induction in the floral buds of ‘Old Blush’ A–E Five floral bud stages of ‘Old Blush’, bar = 5 mm. F. PMC at floral bud stage 1; almost all are polygonal and the nucleoli are distinct; G. PMC at floral bud stage 2, the early stage in meiosis; H. PMC at floral bud stage 3; most are dyad; I. PMC at floral bud stage 4; the dyads and tetrads are roughly half of the total pollens, including a small amount of triads; J. PMC at floral bud stage 5; meiosis is complete and all cells are tetrads, F–J. bar = 10 µm. K. Interim I; L. Late II; M. Terminal II (quadruplex); N. Lagging chromosomes of metaphase I; O. Parallel spindle (pole view); P. Diploid; Q. Triad, K–Q. bar = 5 µm. R–T. 1n natural pollen; U-W. Colchicine-induced 1n pollen; X-Z. Natural 2n pollen; AA-CC. Colchicine-inducted 2n pollen; R,U,X,AA. Pollen pole view (2000 ×5KV); S,V,Y,BB. Pollen equatorial view (2000 × 5KV), bar = 10 µm; T,W,Z,CC. Pollen surface ornamentation (8000 × 5KV), bar = 5 µm. DD-FF.Colchicine-induced 2n pollen of ‘Old Blush’ under different treatments, DD. Induction rate of 2n pollen; EE. Induced 2n pollen vitality; FF. Induction rate of viable 2n pollen.“*”significance testing, *P* ¡0.05; “**”significance testing,* P* ¡0.01, analysis of significant differences in different treatment concentrations.

## Additional Information and Declarations

### Data Availability

The following information was supplied regarding data availability: The raw data are available as Supplemental Files.
