# Supplementary material for: Colchicine induction of ‘Old Blush’ 2n pollen for the hybridization and breeding of tetraploid rose
Source: PeerJ. 2021 Mar 9;9:e11043. doi: 10.7717/peerj.11043 (PMC7953881; doi:10.7717/peerj.11043)
Supplement: Supplemental Information 1 [file peerj-09-11043-s001.docx]

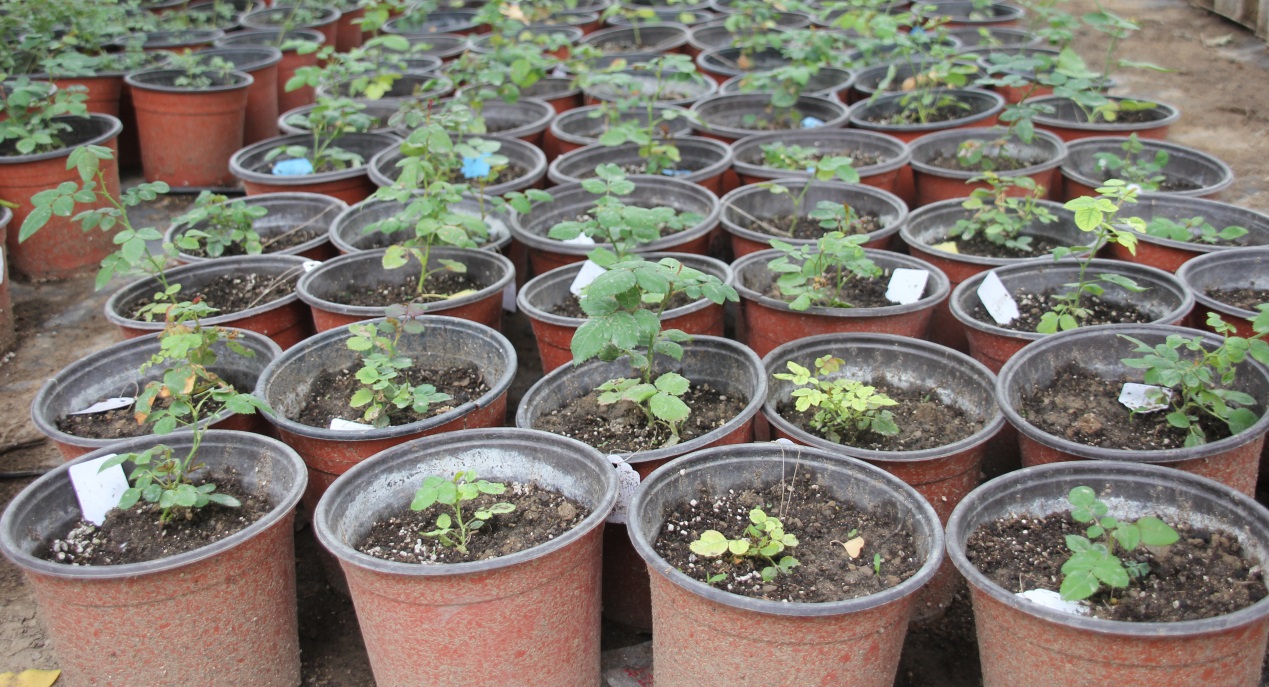


Fig.S1F1 offspring seedlingsnormally growth from seeds of colchicine-induced ‘Old Blush’ 2n pollen pollination on‘Orange Fire’ pistil.


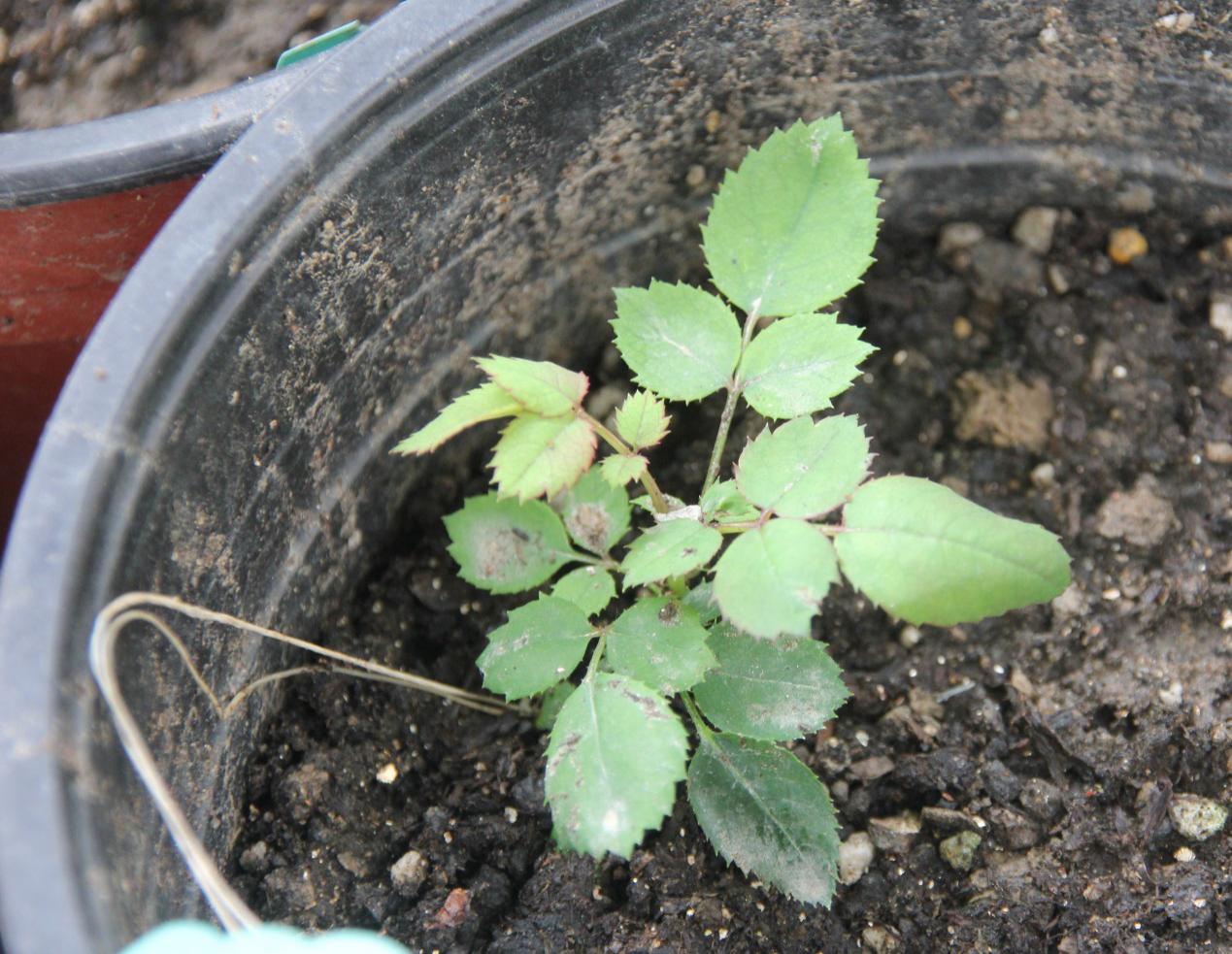


Fig.S2 F1 offspring seedling normally growth from seedof colchicine-induced‘Old Blush’ 2n pollen pollination on ‘Orange Fire’ pistil.


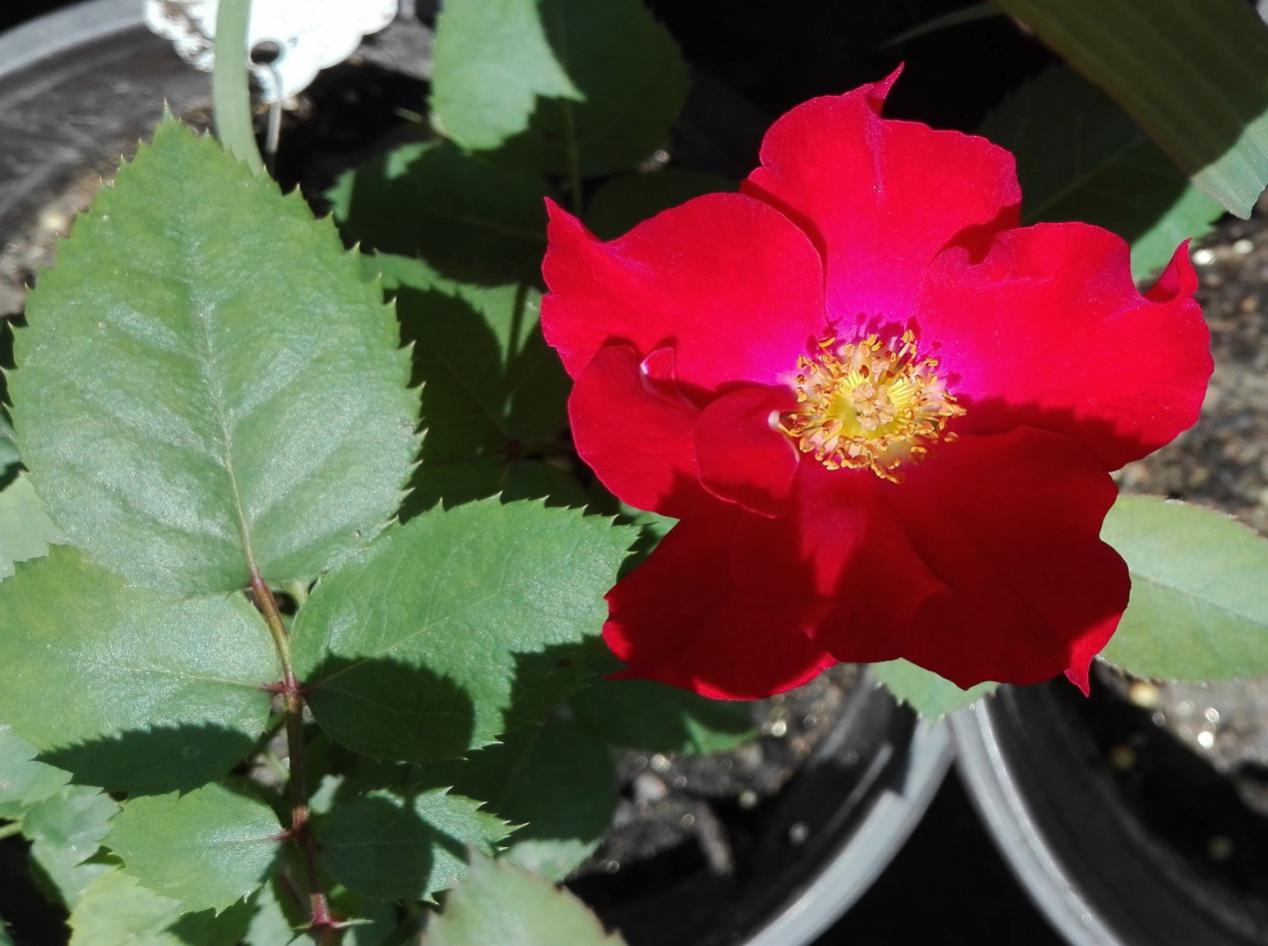


Fig. S3 F1 offspring plant normally flowering of colchicine-induced ‘Old Blush’ 2n pollen pollination on ‘Orange Fire’ pistil.


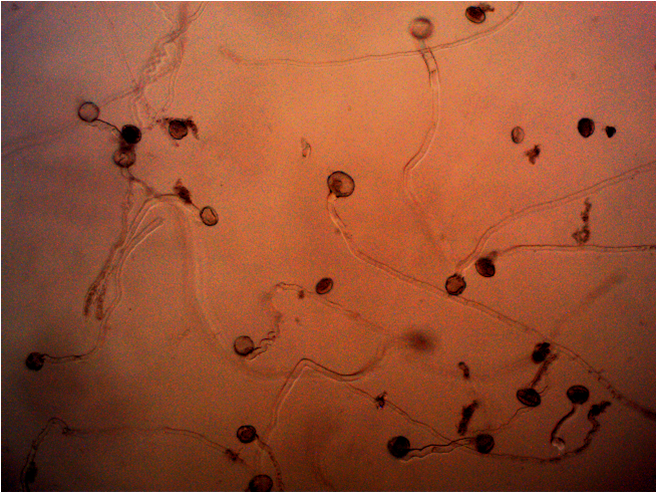


Fig. S4 original images for Figure 2C-colchicine-induced ‘Old Blush’ 2n pollen or colchicine-treated ‘Old Blush’ 1n pollen germination in vitro


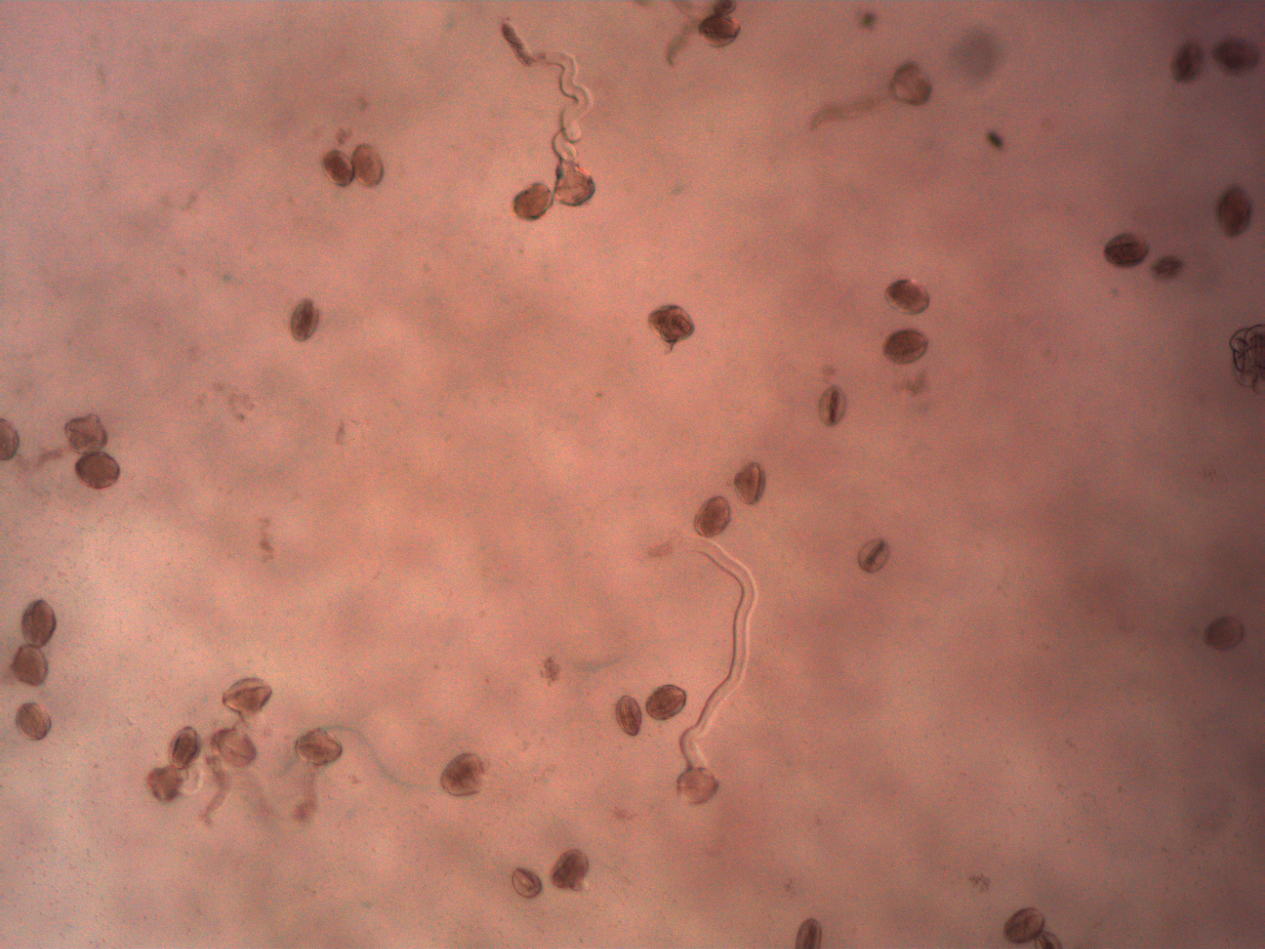


Fig. S5 original images for Figure 2B-natural 1n pollen germination in vitro
