# Supplementary material for: Colchicine induction of ‘Old Blush’ 2n pollen for the hybridization and breeding of tetraploid rose
Source: PeerJ. 2021 Mar 9;9:e11043. doi: 10.7717/peerj.11043 (PMC7953881; doi:10.7717/peerj.11043)
Supplement: Supplemental Information 2 [file peerj-09-11043-s002.doc]

**Table S1. The 21 pairs of rose SSR primers used in paternity test**

| SSR Number | Positive(5’-3’) | Reverse(3’-5’) | target fragment (bp) |
| --- | --- | --- | --- |
| Rw10J19 | GCGAGTTGACGACGAGTT | GGGTGGGCTTCCTTAGTTA | 373 |
| Rw3N19 | CTGGCTGGTTCTCTTTCTG | ATGGGTCGTCGTCGATATG | 125 |
| Rw5D11 | CAGATTCGCCGTAGCCCTTAC | ATCCGAACCCCGACCTGAC | 254 |
| Rw11E5 | GATACCGCGAAGGTGTAGT | GAGTGAAAACTCTGCAATCA | 173 |
| Rw23H5 | AAGCTCTGCCATTGTCCACT | GCCCCTCCAAACTTAACCTC | 125 |
| Rw27A11B | TGTTCCCTTTTAATGAATTAGC | GTTCATCCCTTCAAACCAC | 313 |
| Rw46O8 | ACATGTGGTGCTGTGTTT | GTACAGGCCACTGCTGTC | 307 |
| Rw48N6 | GAGGGCGATCTTCGTATTCTC | GGGGCAATTGAAGGGTTTAG | 272 |
| Rw50N23 | AATTGGTATATTCTGGTAAGTAG | ATAGGAGGGTGATGACTAAC | 567 |
| Rw55C6 | GTGGATTTTCAGAGATACGC | TCACAGACAGGACCACCTAT | 265 |
| Rw61F2 | GTTGGAATTGCAGAGGTGAT | AACTAAAGGCAGGCCACTAA | 250 |
| Rog5 | ATTTTAGTTTCCTAGAGCAGA | ATCGTGGTATTTCATCGG | 141-153 |
| Rog9 | TCCTGAAAACGAAGCCTCC | TTCTCCGCCCTATCCAATG | 136-152 |
| Rog26 | CCGCACCCTACCTAAAAT | ATTGAATGGTTGAGATGCC | 123-141 |
| Rog27 | CTGGTGGAATATTTCATGC | TGGAATACAGAATCAAGAACA | 201-221 |
| C172 | ACAACACCAACTAGAACTTGAGC | GCTCAACAGCAACAACCTCA | 138-158 |
| C187 | TGTGCCTCGAGAGGTTTCTT | GTCAGCTGAAGCACTGGTGA | 197-209 |
| H9B01 | TCTGGTTGGTGATGATGAGC | CGTAAACCATTCCGTGTTCC | 232 |
| H10D03 | CAATTCAAAACCACCGCTCT | CGCAGAGTCAACGAACCATA | 222 |
| H20D08 | TTCGGCTCTCTTCTCTGCTC | GACATTACAGCGACGAAGCA | 240 |
| H22E04 | GACATCACCACCACCACAAG | AACCAAGGTTTCCAGTTCCA | 241 |

**Table S2.** Effect of colchicine treatment with different concentration and treatment time on ‘Old Blush’ buds

| NO. | Treatment  time (h) | Treatment  concentrations  (%) | Flower survival rate (%) | 2n induction  rate (%) | Pollen viability (%) | | Induction rate of viable 2n pollen (%) |
| --- | --- | --- | --- | --- | --- | --- | --- |
|  |  |  |  |  | 1n | 2n |  |
| 1 | 0 | 0 | 100.0a | 1.01i | 88.5a | 100.0a | 1.01f |
| 2 | 24 | 0 | 100.0a | 1.01i | 84.2b | 100.0a | 1.03f |
| 3 | 24 | 0.25 | 88.9b | 5.20h | 81.1c | 75.0b | 3.90b |
| 4 | 24 | 0.50 | 65.0d | 15.83a | 78.1d | 27.2d | 4.31a |
| 5 | 24 | 0.75 | 52.9f | 6.65g | 68.0g | 16.9e | 1.12f |
| 6 | 24 | 1.00 | 45.0g | 9.89e | 57.7i | 1.6g | 0.16g |
| 7 | 48 | 0 | 100.0a | 0.97i | 82.5b | 100.0a | 0.97f |
| 8 | 48 | 0.25 | 65.0d | 7.92f | 74.7e | 50.0c | 3.96b |
| 9 | 48 | 0.50 | 56.3e | 10.25de | 74.0e | 26.8d | 2.75d |
| 10 | 48 | 0.75 | 42.1h | 8.49f | 64.5h | 12.3ef | 1.04f |
| 11 | 48 | 1.00 | 40.0i | 11.50c | 55.8j | 1.2g | 0.14g |
| 12 | 72 | 0 | 85.0c | 0.99i | 80.6c | 100.0a | 0.99f |
| 13 | 72 | 0.25 | 57.9e | 6.65g | 70.9f | 25.0d | 1.66e |
| 14 | 72 | 0.50 | 40.9hi | 12.80b | 55.1j | 25.0d | 3.20c |
| 15 | 72 | 0.75 | 40.0i | 10.91cd | 58.4i | 10.0f | 1.09f |
| 16 | 72 | 1.00 | 20.0j | 12.77b | 50.6k | 0.8g | 0.10g |

**Table S3.** Analysis of variance (ANOVA) of the colchicine-induced 2n pollen in ‘Old Blush’

| Variance | SS | | df | | MS | | F_Value | | P_Value | | Critical value | |
| --- | --- | --- | --- | --- | --- | --- | --- | --- | --- | --- | --- | --- |
|  | Induction rate | Induction rate of viable2n pollen | Induction rate | Induction rate of viable 2n pollen | Induction rate | Induction rate of viable 2n pollen | Induction rate | Induction rate of viable 2n pollen | Induction rate | Induction rate of viable 2n pollen | Induction rate | Induction rate of viable 2n pollen |
| Concentration | 249.55 | 19.86 | 5 | 5 | 49.9106 | 6.34 | 6.67** | 14.33** | 0.00186 | 0.00535 | e2.90 | 2.90 |
| Time | 179.59 | 3.87 | 3 | 3 | 59.86 | 0.60 | 7.99** | 1.35 | 0.00204 | 0.20664 | 3.29 | 3.29 |
| Error | 112.29 | 11.27 | 15 | 15 | 7.49 | 0.44 |  |  |  |  |  |  |
| Total | 541.43 | 30.10 | 23 | 23 |  |  |  |  |  |  |  |  |

Notes. *significance testing, *P*<0.05 **significance testing, *P*<0.01

**Table S4. Floral bud survival rate following colchicine treatment**

| NO. | Time (h) | Concentration (%) | Number of pollinated floral buds | Number of floral buds | Survival number of floral buds | Survival rate of floral buds (%) |
| --- | --- | --- | --- | --- | --- | --- |
| 1 | 0 | 0 | 20 | 20 | 20 | 100.00 |
| 2 | 24 | 0 | 20 | 20 | 20 | 100.00 |
| 3 | 24 | 0.25 | 20 | 18 | 16 | 88.90 |
| 4 | 24 | 0.50 | 20 | 20 | 13 | 65.00 |
| 5 | 24 | 0.75 | 20 | 17 | 9 | 52.90 |
| 6 | 24 | 1.00 | 20 | 20 | 9 | 45.00 |
| 7 | 48 | 0 | 20 | 20 | 20 | 100.00 |
| 8 | 48 | 0.25 | 20 | 20 | 13 | 65.00 |
| 9 | 48 | 0.50 | 20 | 16 | 9 | 56.30 |
| 10 | 48 | 0.75 | 20 | 19 | 8 | 42.10 |
| 11 | 48 | 1.00 | 20 | 20 | 8 | 40.00 |
| 12 | 72 | 0 | 20 | 20 | 17 | 85.00 |
| 13 | 72 | 0.25 | 20 | 19 | 11 | 57.90 |
| 14 | 72 | 0.50 | 25 | 22 | 9 | 40.90 |
| 15 | 72 | 0.75 | 20 | 20 | 8 | 40.00 |
| 16 | 72 | 1.00 | 20 | 20 | 4 | 20.00 |

**Table S5.** ANOVA of the floral bud survival rate following colchicine treatment

| Variance | SS | df | MS | F-value | P-Value | Critical value |
| --- | --- | --- | --- | --- | --- | --- |
| Concentration | 6667.6267 | 4 | 1666.9067 | 53.199432** | 8.367E-06 | 3.8378534 |
| Time | 1170.5813 | 2 | 585.29067 | 18.679589** | 0.0009676 | 4.4589701 |
| Error | 250.66533 | 8 | 31.333167 |  |  |  |
| Total | 8088.8733 | 14 |  |  |  |  |

Notes. *significance testing, *P*<0.05; **significance testing, *P*<0.01

**Table S6. Induction rate of viable 2n pollen**

| Concentration (%) | Time (h) | | | | | | | | | |
| --- | --- | --- | --- | --- | --- | --- | --- | --- | --- | --- |
|  | 0 | | 24 | | | 48 | | 72 | | |
|  | AVG. | S.D. | AVG. | | S.D. | AVG. | S.D. | AVG. | | S.D. |
| 0 | 100 | 0 | 100 | 0 | | 100 | 0 | 100 | 0 | |
| 0.10 | 100 | 0 | 100 | 0 | | 83 | 8.88819 | 80 | 7.54983 | |
| 0.25 | 100 | 0 | 75 | 5 | | 50 | 7.2111 | 25 | 4.66047 | |
| 0.5 | 100 | 0 | 27.2 | 4.41928 | | 26.8 | 2.69072 | 25 | 4.71593 | |
| 0.75 | 100 | 0 | 16.9 | 2.51595 | | 12.3 | 1.79544 | 10 | 0 | |
| 1.00 | 100 | 0 | 1.6 | 0.58129 | | 1.2 | 0.19 | 0.8 | 0.09165 | |

**Table S7.** Survival rate of ‘Old Blush’ floral buds.

| Concentration (%) | Time (h) | | | | | | | | | | |
| --- | --- | --- | --- | --- | --- | --- | --- | --- | --- | --- | --- |
|  | 0 | | 24 | | | 48 | | | 72 | | |
|  | AVG. | S.D. | AVG. | | S.D. | AVG. | | S.D. | AVG. | | SD. |
| 0 | 100 | 0 | 100 | 0 | | 100 | 0 | | 85 | 5 | |
| 0.25 | 100 | 0 | 88.9 | 3.91535 | | 65 | 5.56776 | | 57.9 | 2.45153 | |
| 0.50 | 100 | 0 | 65 | 6.245 | | 56.3 | 5.48361 | | 40.9 | 5.01199 | |
| 0.75 | 100 | 0 | 52.9 | 6.55973 | | 42.1 | 3.0348 | | 40 | 3.747 | |
| 1.00 | 100 | 0 | 45 | 0.1 | | 40 | 1.81934 | | 20 | 1.0583 | |

**Table S8.** Seed setting rate of ‘Orange Fire’ (♀) × colchicine-induced ‘Old Blush’ 2n pollen (♂)

| Pollen type | Seed setting rate(%) | S.D. |
| --- | --- | --- |
| Natural pollen | 63.61 | 3.18 |
| Induced pollen | 55.57 | 2.78 |
